# Risk of secondary cancers from scattered radiation during intensity-modulated radiotherapies for hepatocellular carcinoma

**DOI:** 10.1186/1748-717X-9-109

**Published:** 2014-05-08

**Authors:** Dong Wook Kim, Kwangzoo Chung, Weon Kuu Chung, Sun Hyun Bae, Dong Oh Shin, Seongeon Hong, Sung Ho Park, Sung-Yong Park, Chae-Seon Hong, Young Kyung Lim, Dongho Shin, Se Byeong Lee, Hyun-ho Lee, Jiwon Sung, Myonggeun Yoon

**Affiliations:** 1 Department of Radiation Oncology, KyungHee University Hospital at Gangdong, Seoul, Korea; 2 Deparment of Radiation Oncology, Samsung Medical Center, Seoul, Korea; 3 Department of Radiation Oncology, KyungHee University Medical Center, Seoul, Korea; 4 Department of Neurosurgery, Ulsan University Hospital, Ulsan, Korea; 5 Proton Therapy Center, McLaren Cancer Institute, Flint, USA; 6 Proton Therapy Center, National Cancer Center, Ilsan, Korea; 7 Department of Radiological Science, College of Health Science, Korea University, Jeongneung 3-dong, Seongbuk-gu, Seoul, Korea

**Keywords:** HCC, IMRT, VMAT, Tomotherapy, Radiophotoluminescence, OED, EAR, ERR, LAR

## Abstract

**Purpose:**

To evaluate and compare the risks of secondary cancers from therapeutic doses received by patients with hepatocellular carcinoma (HCC) during intensity-modulated radiotherapy (IMRT), volumetric arc therapy (VMAT), and tomotherapy (TOMO).

**Methods:**

Treatments for five patients with hepatocellular carcinoma (HCC) were planned using IMRT, VMAT, and TOMO. Based on the Biological Effects of Ionizing Radiation VII method, the excess relative risk (ERR), excess absolute risk (EAR), and lifetime attributable risk (LAR) were evaluated from therapeutic doses, which were measured using radiophotoluminescence glass dosimeters (RPLGDs) for each organ inside a humanoid phantom.

**Results:**

The average organ equivalent doses (OEDs) of 5 patients were measured as 0.23, 1.18, 0.91, 0.95, 0.97, 0.24, and 0.20 Gy for the thyroid, lung, stomach, liver, small intestine, prostate (or ovary), and rectum, respectively. From the OED measurements, LAR incidence were calculated as 83, 46, 22, 30, 2 and 6 per 10^4^ person for the lung, stomach, normal liver, small intestine, prostate (or ovary), and rectum.

**Conclusions:**

We estimated the secondary cancer risks at various organs for patients with HCC who received different treatment modalities. We found that HCC treatment is associated with a high secondary cancer risk in the lung and stomach.

## Introduction

Hepatocellular carcinoma (HCC), the most common primary cancer of the liver, is a malignant disease that causes death within a few months, unless it is treated appropriately [1,2]. Surgical resection is the standard treatment for HCC, but approximately 80% of cases are unresectable, generally because of preexisting hepatic dysfunction associated with cirrhosis or the multifocality of its presentation [3]. Transcatheter arterial chemoembolization (TACE), percutaneous ablation [4,5], and radiation therapy (RT) [6,7] have been used for patients with unresectable HCC, but the standard treatment modality for primary HCC has not yet been established. Only TACE has been proven to provide a survival benefit in a phase III study of advanced-stage disease [8]. In the past, the role of RT for HCC has been limited because of the low tolerance of the liver to RT and the risks of radiation-induced liver disease [9]. However, RT treatments have tended to shift from palliative to cure-oriented therapies with each new development in RT techniques, such as intensity-modulated radiotherapy (IMRT) [10-16] (including volumetric-modulated arc therapy [17,18]), helical tomotherapy (TOMO) [19-24] and particle therapy [25-27].

When tumors are exposed to the high doses that are prescribed for a definitive or palliative goal, the surrounding normal tissues are generally exposed to intermediate doses because of the primary radiation in the beam path. Therefore, the treatment planning is optimized to identify the option that best satisfies two conflicting priorities: reducing the dose that the surrounding normal organ is exposed to, and focusing the prescription dose into a target volume. However, out-of-field exposure is another issue of concern; during radiation treatment, the rest of the body is also exposed to low doses because therapeutic radiation in out-of-field region where is all tissues without the trans-axial planed of PTV. Therefore, it is also important to measure the exposed dose for normal organs in out-of-field regions, as well as the corresponding cancer risk.

To date, there have been many measurements of secondary scattered dose and many assessments of secondary cancer risk [28-34]. These studies reflect concerns that the secondary cancer risk may be increased by IMRT compared with that by 3D-CRT because IMRT uses more fields and monitor units, which cause a higher whole-body exposure to leakage radiation. It has been reported that IMRT induces almost twice the incidence of second malignancies that is associated with 3D-CRT [28-34]. Yoon et al. have investigated the secondary scattered radiation doses of IMRT and proton therapy for patients with lung and liver cancer [31]. They presented secondary scattered dose measurements for IMRT at 20–50 cm from the isocenter, which ranged from 5.8 to 1.0 mGy per 1 Gy of the target volume dose (Gray [Gy] is the SI unit of therapeutic absorbed dose). In a previous study, we reported organ equivalent dose (OED) measurements for patients with stage III non-small cell lung cancer [30]. The mean values of the relative OEDs of secondary doses from VMAT and TOMO, which were normalized by IMRT, ranged from 88.63% to 41.59%.

In this study, we compared the risks of secondary cancer from out-of-field and in-field radiation for three treatment modalities, using the concept of OED for radiation-induced cancer in patients with primary HCC.

## Methods and materials

### Patient data and treatment planning

We randomly selected five HCC patients who were to be treated with double arc VMAT at Kyunghee University Hospital, Gangdong. Each of these patients had undergone treatment planning computed tomography (CT) (Brilliance CT Big Bore Oncology; Philips Medical System, Amsterdam, The Netherlands) to identify targets and normal neighboring organs. Eclipse (Varian Medical Systems, Palo Alto, CA, USA) and Hi-Art (TomoTherapy, Madison, WI, USA) planning systems were used to plan IMRT, VMAT, and TOMO for these patients. As shown in Table 1, the patient group consisted of four male patients and one female patient. The ages of the patients ranged from 42 to 62 years, with a mean age of 53 years. All patients had primary HCC with a single target, and planning target volumes (PTVs) varied from 60 to 2112 cc.

**Table 1:** Patient information

**ID**	**Sex**	**Age**	**Disease**	**Stage**	**PTV volume (cc)**	**Prescription dose (Gy)**
**1**	Male	62	HCC	III	483	55.0
**2**	Male	54	HCC	I	60	66.0
**3**	Male	59	HCC	III	421	52.5
**4**	Female	49	HCC	IV	2112	60.0
**5**	Female	42	HCC	IV	214	72.0

**Figure 1:**
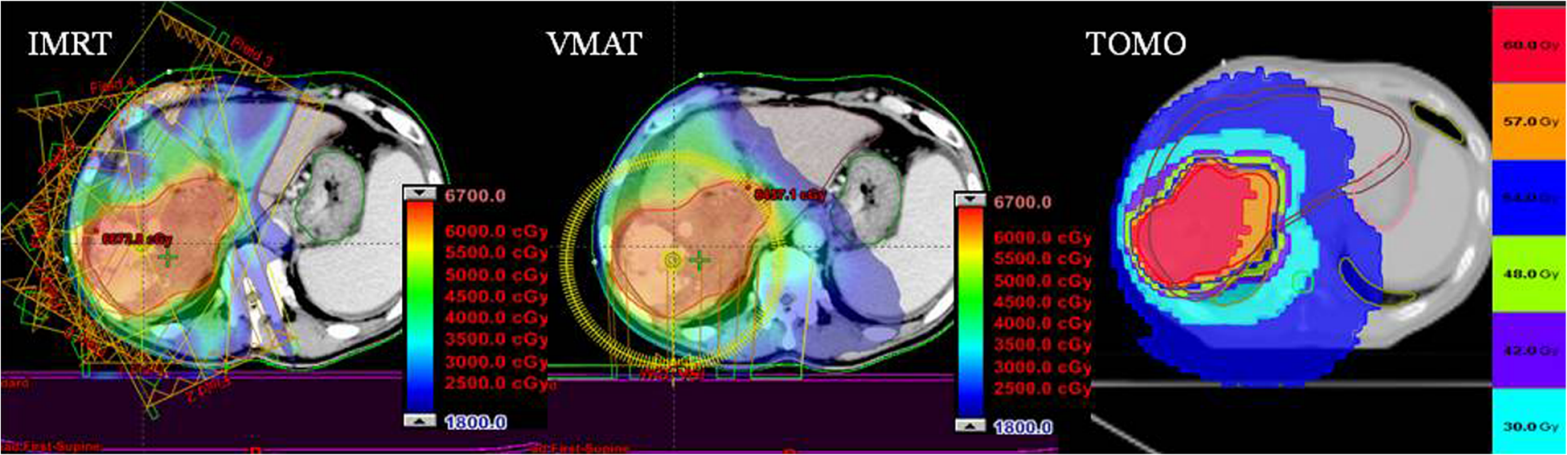
**Patient 4’s dose distribution for different modalities: IMRT, VMAT, and TOMO.** The prescription dose was 62.5 Gy in 25 fractions.

The targets were defined in accordance with Report 50 of the International Commission on Radiation Units and Measurements (ICRU 50). A four-dimensional CT (4DCT) image was obtained during the CT scan using a Philips Brilliant Big Bore CT with a Varian real-time patient monitoring system (RPMS). Particularly, the gross tumor volume (GTV) encompassed all detectable tumors that were observed in the CT scans. The clinical target volume (CTV) included the GTV with a margin for the micro tumor-cell region. The planning target volume (PTV) included the CTV plus a 7–10 mm margin. Each patient received a total dose of 52.5–72.0 Gy to the PTV (using different fractionation schemes) at the isocenter. The prescribed dose was specified at the ICRU reference point (isocenter) of the PTV. All treatment plans used eight beams for IMRT, double arcs for VMAT, and a helical beam for TOMO. 10 MV beam was used for IMRT and VMAT, and 6 MV beam was used for TOMO planning. Examples of the plans are presented in Figure 1, which shows patient 1’s treatment plans for each of the modalities (IMRT, VMAT, and TOMO).

### Calibration of the radiophotoluminescence glass dosimeter

In this study, we used a commercially available radiophotoluminescence glass dosimeter (RPLGD; GD-302 M, Asahi Techno Glass Co., Japan) for dose measurements [35-37]. For these RPLGDs, the absorbed dose was proportional to the light signal (500–700 nm) from the irradiated dosimeter when it was exposed to 365-nm mono-energetic laser light. At energies >200 keV, RPLGDs have a reliable reproducibility of approximately 1% and relatively low energy dependency compared with themoluminescence dosimeters (TLDs) [35-37]. In addition, RPLGDs have a relatively small incident-beam angular dependency and a low toxicity inside the human body compared with TLDs or optically stimulated luminescence dosimeters (OSLDs) [38-40]. Our RPLGDs had a rod-like shape with a diameter of 1.5 mm and a length of 8.5 mm.

RPLGDs were calibrated by measuring the response of each detector after being exposed to a 10 × 10 cm^2^ open field photon beam at the depth of the maximum dose in water-equivalent solid phantom, with a 100-cm source-to-surface distance (SSD) and the absorbed dose at the calibration point was sat as 1 cGy per one monitor units (MU). The reproducibility of the RPLGDs was estimated by calculating the standard deviation of dose measurements that were taken when the same detector was exposed to the photon beam three times. Additionally, the deviations of each RPLGD detector were measured to characterize the RPLGDs.

### Measurement of therapeutic dose during IMRT, VMAT, and Tomotherapy treatment

**Figure 2:**
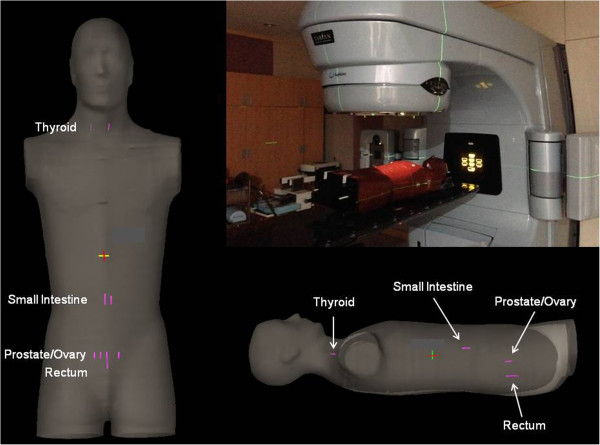
**The setup for the therapeutic dose measurement with a humanoid phantom.** To measure the organ doses in the out-of-field region, two or three RPLGDs were inserted at the positions of interesting organs inside the humanoid phantom: the thyroid, small intestine, prostate/ovary, and rectum.

The treatment beams for IMRT, VMAT, and TOMO were delivered to the humanoid phantom (RANDO® Phantom; The Phantom Laboratory, Salem, NY, USA) using the same patient setup, and the PTV exposure dose was set at 10 Gy. Therapeutic radiation at in-field/out-of-field was assessed by measuring the dose in the RPLGDs at each organ inside the humanoid phantom. These measurements were performed using two to four RPLGDs set in the humanoid phantom at the location of the thyroid, small intestine, prostate/ovary, and rectum, as presented in Figure 2. For the organs adjacent to the PTV (lung, stomach, and normal liver), the doses were estimated using the dose-volume histograms (DVH) of each treatment plan, instead of being measured with RPLGDs. For the accuracy of dos calculation of ECLIPS Analytical Anisotropic Algorithm (AAA) [41,42] and TOMO Hi-Art [43,44], several previous studies report less than 3% of uncertainty in dose calculation in-field region. Some studies reported the our-of-field dose calculation around 50% where the region of iso-dose is less than 10% [45-47]. Therefore, the uncertainty of dose in DVH of the stomach and normal liver were less than 3% because most of volume of these organs covered large than 10% of prescription dose but the uncertainty of lung dose could be large as 50% of organ dose.

### Cancer incidence risk estimation attributable to secondary doses

The lifetime attributable risk (LAR) of incidence for a person who is exposed to radiation dose *D* at age *e*, can be expressed as below (based on the Biological Effects of Ionizing Radiation [BEIR] VII report),

(1) $$\mathrm{LAR}\left(D.e\right)=\int_{e+L}^{100}M\left(D,e,a\right)\times S\left(a\right)/S\left(e\right)\mathrm{da}$$

where *M(D, e, a)* is the excess absolute risk at attained age *a* from exposed age *e*, S(*a*)/S(*e*) is the ratio of the probability of surviving at age *a* and *e*, and L is the latent period (5 y for solid cancers) (National Research Council 2006).

The excess absolute risk (EAR) and excess relative risk (ERR) are modeled according to BEIR VII as functions of sex, age at exposure, and attained age, as below,

(2) $$\mathrm{EAR}\left(x,e,a\right)\;\mathrm{or}\;\mathrm{ERR}\left(x,e,a\right)=\beta_{s}D\;\exp\left(\mathrm{\gamma e}*\right){\left(a/60\right)}^{\eta }$$

where β, γ, η are model parameters, *e* is the age at exposure, e* = (min(*e*,30)-30)/10 and *a* is the attained age. The attained age was arbitrarily set at 20 y after exposure. Table 2 shows the parameter values for preferred risk models in BEIR VII (National Research Council 2006). EAR and ERR of the small intestine were calculated by using the parameters of the colon in Table 2.

**Table 2:** **Parameters for preferred risk incidence models in BEIR VII**^**a**^

**Cancer**	**ERR model**				**EAR model**			
	**β**_**M**_	**β**_**F**_	**γ**	**η**	**β**_**M**_	**β**_**F**_	**γ**	**η**
**Thyroid**	0.53	1.05	−0.83	0.00	Not used			
**Lung**	0.32	1.40	−0.30	−1.40	2.30	3.40	−0.41	5.20
**Stomach**	0.21	0.48	−0.30	−1.40	4.90	4.90	−0.41	2.80
**Liver**	0.32	0.32	−0.30	−1.40	2.20	1.00	−0.41	4.10
**Colon**	0.63	0.43	−0.30	−1.40	3.20	1.60	−0.41	2.80
**Bladder**	0.50	1.65	−0.30	−1.40	1.20	0.75	−0.41	6.00
**Prostate**	0.12	-	−0.30	−1.40	1.20	-	−0.41	2.80
**Ovary**	-	0.38	−0.30	−1.40	-	0.70	−0.41	2.80

^a^From Table Twelve–Two (National Research Council 2006).

The organ equivalent dose (OED) calculation was based on a plateau dose–response model, and is inserted as D in equation (2) and expressed as below,

(3) $$\mathrm{OED}=\frac{1}{V}\sum_{i}V_{i}\left(\frac{1-\exp\left(-\delta D_{i}\right)}{\delta }\right)$$

where *V* is the whole volume, *V*_*i*_ is a volume element, and *D*_*i*_ is the absorbed dose element. In this model, parameters such as δ are used to determine the dose–response curve for specific organs, as presented in Table 3.

**Table 3:** Organ equivalent dose (Gy) per prescription dose at each organ

**Organ**	**δ**	**Modality\ID**	**1**	**2**	**3**	**4**	**5**
**Thyroid**	0.69	IMRT	0.21	0.09	0.28	0.55	0.17
		VMAT	0.15	0.08	0.23	0.47	0.17
		TOMO	0.18	0.14	0.19	0.40	0.11
**Lung**	0.15	IMRT	0.13	0.07	0.18	2.59	0.25
		VMAT	0.13	0.08	0.20	2.87	0.31
		TOMO	1.72	1.65	2.37	3.27	1.89
**Stomach**	1.20	IMRT	0.83	0.82	0.83	0.83	1.08
		VMAT	0.83	0.83	0.83	0.83	1.73
		TOMO	0.83	0.83	0.83	0.83	0.83
**Normal liver**	1.14	IMRT	0.83	0.72	0.83	0.80	1.69
		VMAT	0.83	0.72	0.83	0.83	1.74
		TOMO	0.88	0.86	0.88	0.88	0.88
**Small intestine**	0.26	IMRT	0.67	0.26	0.88	2.04	0.48
		VMAT	0.63	0.29	0.90	2.03	0.69
		TOMO	0.70	0.21	0.88	3.51	0.41
**Prostate/Ovary**	0.73	IMRT	0.25	0.13	0.25	0.59	0.18
		VMAT	0.19	0.07	0.24	0.48	0.17
		TOMO	0.21	0.08	0.22	0.44	0.14
**Rectum**	0.26	IMRT	0.18	0.08	0.22	0.51	0.14
		VMAT	0.15	0.06	0.18	0.41	0.15
		TOMO	0.17	0.06	0.16	0.40	0.12

In this study, we have investigated the OED based cancer incidence risk. The doses and cancer risks were evaluated for thyroid, lung, stomach, normal liver, small intestine, prostate/Ovary and rectum which were provided the parameter values for calculation by preferred risk models in BEIR VII.

## Results and discussion

Table 4 compares the treatment plans for different modalities. For IMRT, eight fields were used. For all fields in each IMRT plan, the total monitor units (MU) per single Gy to the PTV ranged from 312 to 722 MU/Gy. For VMAT, two full arcs were used. For all fields in each VMAT plan, the total MU per single Gy to the PTV ranged from 291 to 346 MU/Gy. For the TOMO plans, the total MU per single Gy to the PTV ranged from 534 to 1865 MU/Gy. More MUs were needs for larger PTV size for IMRT and TOMO but MU of VMAT was not depended significantly for PTV size. Therefore, patient 2 (patient 4) had a relatively lower (higher) MU per Gy than the other patients did, as is evident in Table 4. In addition, the value of MU per Gy depends on the modality. VMAT had a relatively small amount of total treatment MUs (0.7 ± 0.2 times that of IMRT) and no significant dependency with PTV size. TOMO had a comparably large amount of treatment MUs (1.8 ± 0.5 times that of IMRT). As reported in previous studies, VMAT uses less MUs than IMRT and TOMO. Therefore, VMAT facilitates shorter treatment times and fewer MUs that are related to patient immobilization and machine maintenance [30,48,49].

**Table 4:** Treatment planning information

**ID**	**Modality**	**# of fields (or arcs)**	**MU/Gy**
**1**	IMRT	8	543
	VMAT	2	291
	TOMO	n/a	907
**2**	IMRT	8	312
	VMAT	2	346
	TOMO	n/a	534
**3**	IMRT	8	597
	VMAT	2	345
	TOMO	n/a	717
**4**	IMRT	8	722
	VMAT	2	317
	TOMO	n/a	1865
**5**	IMRT	8	384
	VMAT	2	304
	TOMO	n/a	776

**Figure 3:**
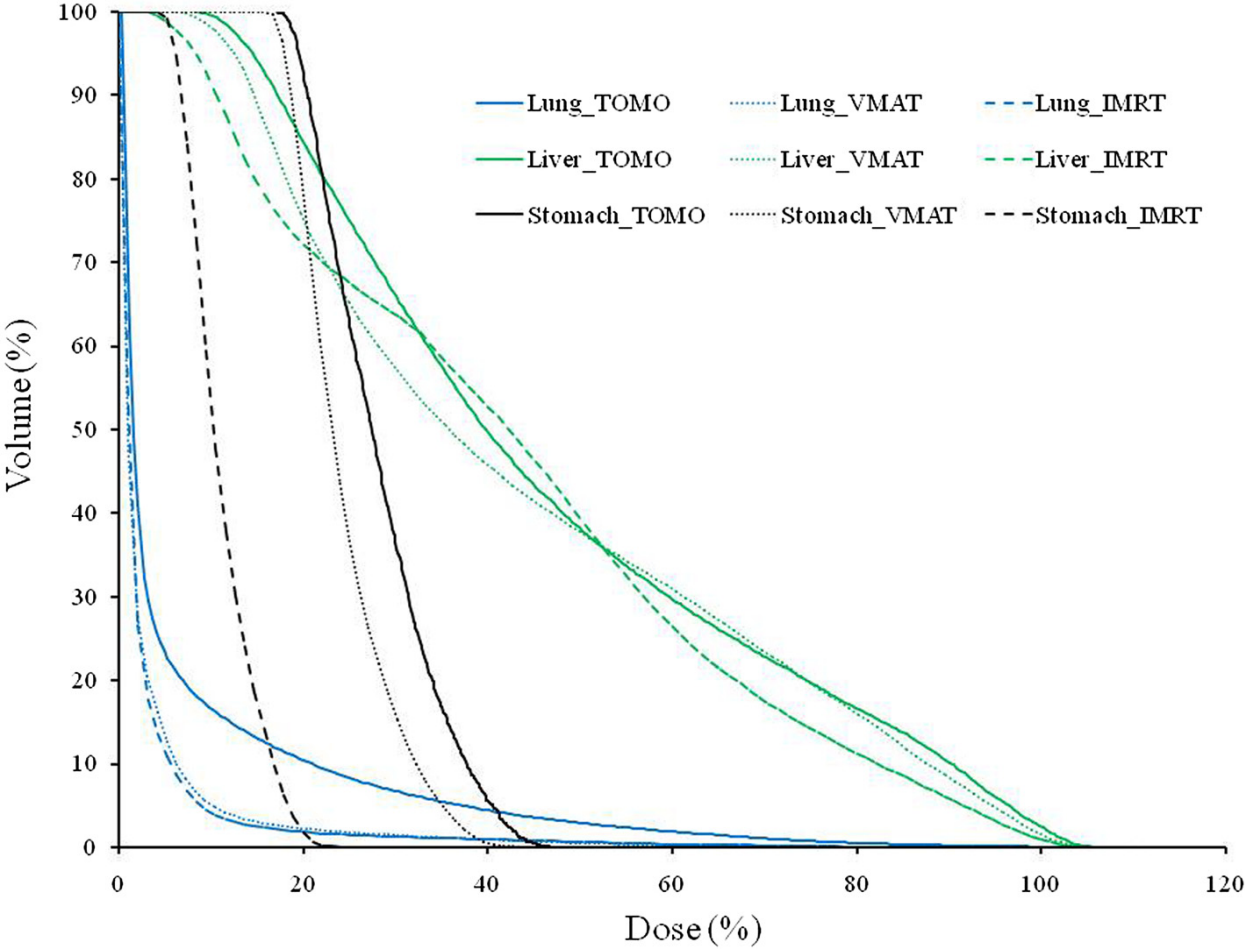
**Dose-volume histogram (DVH) for IMRT (dashed), VMAT (dotted), and TOMO (solid line) plans.** These histograms include dose-volume information of patient 1 for the lung (blue), normal liver (green), and stomach (black).

For each of the five patients, Table 5 presents the dose measurements for IMRT, VMAT, and TOMO in the in-field and out-of-field regions. In-field region is assigned as all tissue within the trans-axial planes of PTV. The mean doses per 1 Gy of therapeutic dose at the thyroid, lung, stomach, normal liver, small intestine, prostate (or ovary), and rectum were 0.5, 3.9, 23.6, 36.3, 1.8, 0.6, and 0.4 cGy/Gy_Rx_ for IMRT; 0.4, 4.2, 28.2, 36.9, 1.9, 0.4, and 0.3 cGy/Gy_Rx_ for VMAT; and 0.4, 8.8, 37.0, 40.4, 4.0, 0.4, and 0.3 cGy/Gy_Rx_ for TOMO, respectively. (Means were taken over the five patients). The measured dose decreased as the distance from the in-field region increased, and increased as the size of PTV. Therefore, patient 2 who have most of small PTV size, gives relatively small measured dose for all organs as shown in Table 5. For the in-field region, the PTV position is also important factor to decide the absorbed dose. In the Table 5, only stomach has not directly depended on the PTV size because of the relative position is more important factor in this case. Therefore the normal liver had the highest organ dose of the measured organs. Although TOMO uses twice the total MUs of other modalities, we did not observe any significant difference in organ dose according to modality for any of the out-of-field organs. Only patient 4 had a greater small intestine dose with TOMO than with other treatment modalities, but differences in planning caused this increase, not the modality itself. Among the in-field region organs, the lung dose was greater with TOMO than with other modalities for the same reason. Figure 3 presents the DVHs for patients 1, and for the lung (blue), stomach (black) and liver (green). As evident in Figure 3, patient 1 had approximately twice the lung dose for the TOMO plan (solid line) than for the IMRT (dashed) or VMAT (dotted) plans. In addition, patient 4 also had a relatively large PTV, which can lead to greater increases in the small intestine dose and the dose-volume distribution for TOMO than for other modalities. Recently, Howell et al. have reported late effects from in-field and out-of-field doses that were sustained during radiation treatment for liver cancer [46]. To obtain in-field and out-of-field doses, they used a DVH and TLD-based dose measurements from a humanoid phantom. The mean doses to the thyroid, stomach, prostate, and rectum were 0.8, 17.5, 0.2, and 0.3 cGy/Gy_Rx_, which are comparable to the measurements in this study: 0.5 ± 0.4, 23.6 ± 16.5, 0.6 ± 0.4, and 0.4 ± 0.3 cGy/Gy_Rx_. The error is calculated from the standard deviation from the dose measurement of five patients and size of error is about 70% of measured value. We expect this error came from the difference of PTV size as mention as above. Therefore, it can be directly compared with Howell et al’s result with PTV size information. In addition, our finding are well matched with previous study by Taddei et al. for the site-specific predicted lifetime risk of second malignant neoplasm (SMN) of HCC at 2010 [50]. They have reported the risks of SMN for thyroid, lung, stomach, normal liver, small bowel and prostate as less than 0.1%, 2.8%, 2.0%, 2.9%, 1.8% and 0.1% which were comparable to our results as less than 0.1%, 2.1%, 2.8%, 3.0%, 1.0% and 0.1% for same site. Both results show good agreements at most of organs. To compare the both results directly, we calculated the risks of SMN by using their calculation skim with our measurements. For five patients, the predicted lifetime risks of normal liver were ranged from 2.6% to 7.2% and we found the risks of SMN become larger when the PTV size increasing.

**Table 5:** The absorbed dose per 1 Gy of therapeutic dose at each organ

**Organ**	**Modality\ID**	**Organ dose per 1 Gy (cGy/Gy)**				
		**1**	**2**	**3**	**4**	**5**
**Thyroid**	IMRT	0.4	0.1	0.6	1.1	0.3
	VMAT	0.3	0.1	0.5	0.9	0.3
	TOMO	0.4	0.2	0.4	0.8	0.2
**Lung**	IMRT	2.6	1.1	5.2	8.8	1.8
	VMAT	2.8	0.1	5.5	10.1	2.4
	TOMO	6.9	3.0	11.6	16.5	6.1
**Stomach**	IMRT	10.5	30.7	49.3	26.0	1.7
	VMAT	23.4	30.5	49.5	30.3	7.3
	TOMO	28.3	46.7	53.3	43.0	13.5
**Normal liver**	IMRT	41.0	20.1	45.2	45.7	29.3
	VMAT	42.7	18.5	38.7	54.1	30.6
	TOMO	47.0	25.3	44.9	49.8	35.0
**Small intestine**	IMRT	1.3	0.4	1.9	4.8	0.7
	VMAT	1.3	0.5	1.9	4.8	1.1
	TOMO	1.4	0.3	1.9	15.7	0.6
**Prostate/Ovary**	IMRT	0.5	0.2	0.5	1.2	0.3
	VMAT	0.4	0.1	0.5	1.0	0.3
	TOMO	0.4	0.1	0.5	0.9	0.2
**Rectum**	IMRT	0.3	0.1	0.4	0.9	0.2
	VMAT	0.3	0.1	0.4	0.7	0.2
	TOMO	0.3	0.1	0.3	0.7	0.2

The in-field region organs were the lung, stomach, and normal liver. The other organs were out-of-field. The doses of in-field region organs were calculated from the dose-volume histogram, and the doses of out-of-field region organs were measured by RPLGD detectors inside a humanoid phantom.

For the absorbed doses of organs in in-field region, the uncertainties were assumed to less than 3% because the absorbed dose values from primary radiation for IMRT, VMAT and TOMO were based on the dose calculation from the radiation treatment planning system (RTPS). The dose measurement uncertainties of organs at out-of-fields region where is mainly contributed by stray radiation was less than 3% for each RPLGD measurement.

For each of the five patients, Table 3 presents OED measurements (or calculation from DVH) for IMRT, VMAT, and TOMO. The mean OEDs per prescription dose at the thyroid, lung, stomach, normal liver, small intestine, prostate (or ovary), and rectum were 0.26, 0.65, 0.88, 0.98, 0.87, 0.28, and 0.22 Gy for IMRT; 0.22, 0.72, 1.01, 0.99, 0.91, 0.23, and 0.18 Gy for VMAT; and 0.21, 2.18, 0.83, 0.87, 1.14, 0.22, and 0.18 Gy for TOMO, respectively. (Means were taken over the five patients). The OED decreased as the distance from the in-field region increased. For the OED measurement at out-of-field region, the OED differences for three different modalities at each organ were less than 10% except patient 2. This result conflicts with the findings of a previous study on lung cancer [30]. In this previous study, we reported that TOMO resulted in lower OEDs than IMRT or VMAT, based on estimations of OED for the thyroid, pancreas, bowel, rectum, and prostate. The main difference between these two studies is the measurement setup and treatment site. In the previous study, RPLGDs were positioned on the treatment table without build-up material. In this study, RPLGDs were inserted into the humanoid phantom at each organ position. Because the previous study could not include the maximum out-of-field dose without the build-up material, 6 MV TOMO provided lower OEDs than IMRT or VMAT (which usually use 6 MV photon beam). For in-field region, the OED of lung was greater with TOMO than other modalities as shown as Table 3 because the absorbed dose of lung with TOMO was relatively higher than other modalities. Because the OED calculation was based on a plateau-response model which is converged to 1/δ with high absorbed dose, the OED values of stomach and normal liver were close to 0.83.

Tables 6 and 7 presents ERR, EAR, and LAR results, which indicate that 59, 45, 21, 27, 2, 7 patients, 65, 46, 21, 27, 2, 6 patients, and 124, 46, 23, 37, 1, 5 patients per 10,000 person for IMRT, VMAT and TOMO will develop radiation-induced cancers of the lung, stomach, normal liver, small intestine, prostate (or ovary), and rectum in the remainder of the lifetime following radiation treatment for HCC. TOMO has relatively higher risk at lung and small intestine comparing to other modalities. The sum of LARs for each site from this study indicates that 1.6%, 1.6% and 2.4% of patients with HCC will develop radiation-induced cancer in the remainder of the lifetime following radiation therapy (RT) by IMRT, VMAT and TOMO. Brenner et al. (2000) studied the secondary cancer risk in prostate cancer patients and estimated the incidences of extra solid tumors after radiotherapy [51]. They reported, among 17,327 persons at risk, 139 extra solid tumors were estimated to be induced by radiation treatment. This indicates that the sum of LARs due to the prostate radiotherapy is approximately 0.27% which is less than the risk with HCC treatment. This may be due to the fact that the exposed age is high and the number of organs at risk is few for prostate cancer treatment compared to HCC treatment. This comparison indicates that the LAR value is critically dependent on the site of cancer and the exposed age.

**Table 6:** Excess relative risk (ERR) and excess absolute risk (EAR) for five patients

**Organ**	**Modality\ID**	ERR (EAR*)				
		**1**	**2**	**3**	**4**	**5**
**Thyroid**	IMRT	0.11	0.05	0.15	0.58	0.18
	VMAT	0.08	0.05	0.12	0.49	0.18
	TOMO	0.10	0.08	0.10	0.42	0.12
**Lung**	IMRT	0.03 (1.47)	0.02 (0.50)	0.04 (1.76)	2.98 (18.22)	0.34 (1.01)
	VMAT	0.07 (1.51)	0.02 (0.53)	0.04 (1.95)	3.31 (22.20)	0.42 (1.26)
	TOMO	0.91 (20.08)	0.39 (11.28)	0.52 (22.77)	3.76 (22.97)	2.53 (7.62)
**Stomach**	IMRT	0.11 (9.76)	0.13 (7.22)	0.12 (8.82)	0.33 (6.04)	0.49 (5.79)
	VMAT	0.44 (9.79)	0.13 (7.28)	0.12 (8.82)	0.33 (6.04)	0.79 (9.29)
	TOMO	0.44 (9.79)	0.13 (7.35)	0.12 (8.82)	0.33 (6.04)	0.38 (4.48)
**Normal liver**	IMRT	0.17(6.59)	0.17 (3.76)	0.18 (5.62)	0.21 (1.42)	0.52 (1.94)
	VMAT	0.44 (6.60)	0.17 (3.75)	0.18 (5.64)	0.22 (1.48)	0.53 (2.00)
	TOMO	0.46 (6.95)	0.20 (4.46)	0.19 (5.95)	0.23 (1.56)	0.27 (1.00)
**Small intestine**	IMRT	0.27 (5.16)	0.12 (1.47)	0.38 (6.08)	0.72 (4.82)	0.20 (0.85)
	VMAT	0.33 (4.85)	0.13 (1.65)	0.38 (6.20)	0.72 (4.80)	0.28 (1.22)
	TOMO	0.37 (5.35)	0.10 (1.20)	0.38 (6.05)	1.24 (8.31)	0.27 (0.71)
**Prostate/Ovary**	IMRT	0.02 (0.07)	0.01 (0.03)	0.02 (0.06)	0.18 (0.61)	0.07 (0.14)
	VMAT	0.10 (0.05)	0.01 (0.01)	0.02 (0.06)	0.15 (0.49)	0.06 (0.13)
	TOMO	0.11 (0.06)	0.01 (0.01)	0.02 (0.05)	0.14 (0.46)	0.05 (0.11)
**Rectum**	IMRT	0.07 (1.36)	0.04 (0.44)	0.09 (1.49)	0.18 (1.20)	0.06 (0.25)
	VMAT	0.08 (1.12)	0.03 (0.35)	0.08 (1.26)	0.14 (0.96)	0.06 (0.26)
	TOMO	0.09 (1.32)	0.03 (0.36)	0.07 (1.13)	0.14 (0.95)	0.05 (0.20)

* EAR: per whole prescribed dose.

**Table 7:** Lifetime attributable risk (LAR) for five patients

**Organ**	**Modality\ID**	LAR*				
		**1**	**2**	**3**	**4**	**5**
**Lung**	IMRT	4.1	3.3	6.9	220.4	18.0
	VMAT	4.3	3.4	7.7	244.3	22.5
	TOMO	56.4	72.6	89.6	277.9	135.3
**Stomach**	IMRT	27.4	46.5	34.7	73.0	102.9
	VMAT	27.5	46.9	34.7	73.0	165.1
	TOMO	27.5	47.3	34.7	73.0	79.5
**Normal liver**	IMRT	18.5	24.2	22.1	17.2	34.4
	VMAT	18.5	24.1	22.2	17.9	35.5
	TOMO	19.5	28.7	23.4	18.8	17.8
**Small intestine**	IMRT	14.5	9.5	23.9	58.4	15.1
	VMAT	13.6	10.6	24.4	58.1	21.6
	TOMO	15.0	7.7	23.8	100.5	12.6
**Prostate/Ovary**	IMRT	0.2	0.2	0.2	7.4	2.5
	VMAT	0.1	0.1	0.2	6.0	2.4
	TOMO	0.2	0.1	0.2	5.5	2.0
**Rectum**	IMRT	3.8	2.8	5.9	14.6	4.4
	VMAT	3.1	2.3	4.9	11.7	4.5
	TOMO	3.7	2.3	4.5	11.5	3.6

*LAR: per 10000 persons.

Although the risk of radiogenic cancer is generally proportional to exposed dose, there are non-negligible uncertainties in the risk model such as the uncertainty in the dose–response relationship for carcinogenesis, uncertainty in the model parameter and etc. The latest report on radiation risk suggested that one cannot choose decisively among the several dose–response models based on the empirical data [17]. This means that there might be large inherent uncertainties in the risk estimation. In addition, there is the systematic uncertainty of applying a risk model for a general U.S. population to international liver cancer patients in our study. This implies that further study on the correlation between dose and secondary cancer risk is needed.

## Conclusion

In this study, we compared secondary cancer risks for patients with HCC. We found that the secondary cancer risk in the out-of-field region depends on the distance from the target volume and the target volume size. Of all the organs that were considered, the lung was subject to the highest risk of radiation-induced cancer after HCC RT.

## Consent

Written informed consent was obtained from the patient for the publication of this report and accompanying images.

## Competing interests

The authors declare that they have no competing interests.

## Authors’ contributions

DWK and MY designed and wrote a first version of the manuscript for this research. WKC, SB and SH provided the patient data and clinical support. KC, CSH, and DOS participated in tomotherapy planning and measurement. YKL, SBL, DHS and SYP contributed in IMRT/VMAT planning and measurement. SHP, HHL and JS participated in the measurements, detector calibrations and EUD calculation. All authors read and approved the final manuscript.
